# The Complete Chloroplast Genome of *Encyclia tampensis* (Orchidaceae): Structural Variation and Heterogeneous Evolutionary Dynamics in Epidendreae

**DOI:** 10.3390/genes16121418

**Published:** 2025-11-28

**Authors:** Bing Liu, Ju Huang, Zishuo Wang, Dong Li, Zhangxi Yuan, Yi Yao

**Affiliations:** 1 College of Landscape Architecture and Horticulture, Yangzhou Polytechnic University, Yangzhou 225009, China; 102129@yzpc.edu.cn (B.L.); huangju214@yeah.net (J.H.); 15206290859@163.com (Z.Y.); 2 School of Grassland Science, Beijing Forestry University, Beijing 100083, China; kingzishuo@bjfu.edu.cn (Z.W.); lidong318@bjfu.edu.cn (D.L.)

**Keywords:** *Encyclia tampensis*, Epidendreae, phylogenetic analysis, structural variation, plastome evolution, selective pressure

## Abstract

Background: The epiphytic orchids of the tribe Epidendreae represent a remarkably evolutionary radiation, yet their phylogenetic relationships and plastome evolutionary dynamics are still not fully resolved. Methods: This study has sequenced, assembled, and annotated the complete chloroplast genome of *Encyclia tampensis*. Through comparative analyses of a curated dataset of 40 Epidendreae plastomes, we investigated codon usage bias, evolutionary selection pressures (Ka/Ks), and phylogenetic relationships. Results: The plastome of *E. tampensis* (160,650 bp) has a typical quadripartite structure, with a significant AT bias (62.09%), and contains 124 annotated genes. Comparative genomic analysis across 40 Epidendreae species revealed substantial plastome size variation (123,455 to 160,650 bp), pronounced small single copy (SSC) contraction in *E. tampensis* (608 bp), and atypical long simple sequence repeats (SSRs) accumulation. Natural selection dominated codon usage, with strongest purifying selection in *rbcL* (average Ka/Ks = 0.205). Phylogenetic analyses confirmed subtribal monophyly and detected evolutionary rate heterogeneity correlated with life history strategies. Conclusions: These results establish that plastome evolution in Epidendreae has been principally driven by structural reorganization through SSC contraction and long SSR accumulation, selective constraints maintaining functional genes under purifying selection, and life history-strategy-mediated evolutionary rate diversification. These processes collectively account for the tribe’s extensive genomic diversity and phylogenetic complexity, thereby providing a theoretical framework for understanding orchid plastome evolution and a molecular basis for the systematic classification and conservation of this economically significant plant group.

## 1. Introduction

Orchidaceae is one of the most phylogenetically derived groups among angiosperms, comprising approximately 899 genera and 28,000 known species [1,2]. Orchids exhibit remarkable diversity and include species of significant ecological and economic importance. They are distributed across a wide range of terrestrial ecosystems, except in extremely cold and arid regions, with their greatest diversity concentrated in tropical areas [3]. Approximately 70% of orchid species grow as epiphytes on other plants, contributing about 20% of the plant diversity in rainforests. These epiphytic orchids provide food and refuge for diverse animals and microorganisms, thereby playing a critical role in maintaining ecosystem health [4,5,6]. The tribe Epidendreae consists predominantly of tropical epiphytes that typically colonize tree surfaces. This growth habit allows them to exploit available space and sunlight, thereby reducing competition with terrestrial plants [7,8]. Through biotic and abiotic interactions associated with the epiphytic niche, this group has undergone substantial species diversification, making it an important system for studying phylogenetic evolution and adaptive radiation within the orchid family [6,9,10].

The delimitation of tribes, subtribes, and genera within Epidendreae has long been controversial, largely due to historical reliance on a limited set of morphological characters. This issue has attracted considerable attention from orchid systematists. The tribe was first described by Kunth in 1815, initially encompassing a broad assemblage of tropical epiphytic orchids [11]. Since then, the circumscription of this tribe has undergone substantial revisions, reflecting a complex history of taxonomic reclassification. From Bentham and Hooker (1883) to Schlechter (1926), and further to Dressler and Dodson (1960), the taxonomic structure at the subtribe level experienced significant adjustments [12,13,14]. Nevertheless, Epidendreae consistently retained a large number of genera. More recent taxonomic revisions, such as those proposed by Dressler and Szlachetko, redefined the tribe under a narrower and more restricted circumscription, though considerable variation persisted in the composition of subtribes assigned to it [15,16]. Based on analysis of the plastid coding *ndhF* gene, Neyland and Urbatsch identified *Sobralia* and *Listera* as lower epidendroids but did not resolve circumscriptions at tribal and subtribal levels [17]. Freudenstein and Rasmussen conducted the first morphological cladistic analysis of the entire Orchidaceae, demonstrating that members of Epidendreae *sensu* Dressler (1993) were distributed across multiple distinct clades. In a comprehensive molecular study based on the plastid gene *rbcL*, Cameron achieved well-resolved delineations at the subfamilial level; however, their analysis provided limited resolution at tribal and subtribal ranks [15,18,19]. Their work led to the division of epidendroids into a ‘lower’ grade and a set of ‘higher’ clades. Another phylogenetic analysis, using mitochondrial DNA data from the *nad1* gene intron, suggested that Epidendreae comprises Laeliinae, Pleurothallidinae, Calypsoeae, and *Bletia* [20]. A phylogenetic assessment of Laeliinae, the largest subtribe within Epidendreae, utilized 295 ITS sequences to evaluate generic delimitations and infer species relationships. The majority of the recovered species groups corresponded to previously recognized taxa at the subgeneric level; however, several genera, including *Encyclia*, *Cattleya*, and *Laelia*, were identified as polyphyletic [21].

*Encyclia*, which ranges from Florida and Mexico to southeastern Brazil, is the second largest genus in subtribe Laeliinae, surpassed in diversity only by *Epidendrum* [22,23,24,25]. The genus currently comprises 213 species, among which 179 have been formally described, and 25 are likely of hybrid origin [26]. *Encyclia* was first established by William J. Hooker in 1828 but was shortly subsumed into a broadly defined *Epidendrum* by Lindley and reduced to subgenus rank [27,28]. Owing to a suite of morphological features that clearly distinguish it from its relatives, taxonomic efforts to reinstate *Encyclia* as a clearly delimited genus were pursued by numerous systematists, from Schlechter to Dressler [29,30]. In 1996, Withner, in his systematic monograph on the Laeliinae, proposed a revised circumscription that aligned more closely with Hooker’s original concept, providing a critical refinement for the genus [31]. Due to the considerable disparity in floral morphology among groups within *Encyclia*, which exceeds the variation observed among many genera in the Laeliinae subtribe, several formerly included groups—such as *Prosthechea*, *Euchile* (later subsumed into *Prosthechea*),and *Dinema*—were subsequently reinstated as separate genera [32,33,34,35,36]. Consequently, the number of species within *Encyclia* has been reduced from a peak of 421 to its current delineation [32,33,37]. In summary, due to limited material availability and an insufficient understanding of phenotypic variation, the classification of the tribe Epidendreae remains contentious. This has resulted in an unstable taxonomic framework that currently lacks consensus.

The chloroplast genome (plastomes) has emerged as a pivotal genetic resource for reconstructing plant phylogenies, species identification, and taxonomic delineation. This prominence stems from several inherent advantages: a highly conserved structure and slow evolutionary rate among most higher plants, resulting in high sequence similarity among closely related species; a significantly smaller size compared to the nuclear genome, which facilitates extraction, purification, and cost-effective sequencing; a rich source of phylogenetic information wherein both sequence and structural variations serve as reliable markers for inferring evolutionary relationships; and predominantly uniparental inheritance and general single-copy nature of genes (excluding the inverted repeat regions), which ensures orthologous comparisons across species [38,39,40,41]. Furthermore, non-coding regions of the plastomes, particularly the hypervariable regions, have proven to be ideal for developing high-resolution DNA barcodes and enabling precise species discrimination [42]. Consequently, leveraging the unique properties of the plastomes provides a comprehensive and clarifying perspective for resolving the complex phylogenetic relationships within the Orchidaceae.

Previous studies utilizing plastome data have successfully reconstructed a consistent and robust phylogenetic framework for the genus *Epidendrum* and its allies, confirming the monophyly of *Epidendrum* [43]. Nonetheless, existing research exhibits several limitations, including insufficient taxonomic sampling, a superficial understanding of the mechanistic drivers underlying observed genomic patterns, and a lack of clear links between plastome architecture and environmental adaptation. To provide a focused and representative case, we selected *Encyclia tampensis*, which is a widely cultivated butterfly orchid native to Florida and the Bahamas, as a key study subject [44]. Therefore, this study will conduct a comprehensive analysis of complete plastomes from *E. tampensis* and across the entire tribe Epidendreae to address these gaps. Our specific objectives are to: (1) systematically characterize the structural features and codon usage bias (CUB) patterns of the Epidendreae plastomes; (2) evaluate the relative contributions of mutational pressure and natural selection in shaping the CUB of Epidendreae using neutrality plot and ENC-plot analyses; and (3) compare structural variations in plastomes across Epidendreae species occupying distinct ecological niches to elucidate the molecular mechanisms underpinning adaptive evolution.

## 2. Materials and Methods

### 2.1. Plant Material, DNA Extraction, and Quality Assessment

Fresh leaf samples of *E. tampensis* were collected from Zengcheng District, Guangzhou, Guangdong Province, China. A voucher specimen was deposited under accession number CS675-004R0002. Healthy young leaves were promptly dried in silica gel and transported to the laboratory for DNA extraction. All collection procedures complied with relevant institutional and national guidelines.

Total genomic DNA was isolated from approximately 100 mg of dried leaf tissue using a modified cetyltrimethylammonium bromide (CTAB) protocol, adapted from established methodologies [45]. The quality and concentration of the extracted DNA were evaluated using a NanoDrop 2000 spectrophotometer (Thermo Fisher Scientific, Waltham, MA, USA) and by agarose gel electrophoresis. Only high-quality DNA samples (OD_260_/_280_ = 1.8~2.0, OD_260_/_230_ ≥ 2.0) were selected for downstream library construction [46].

### 2.2. Genome Sequencing and Plastome Assembly

Whole-genome sequencing libraries were prepared from approximately 1 μg of high-quality genomic DNA using the Illumina TruSeq DNA PCR-Free Library Preparation Kit, following the manufacturer’s instructions. Genomic DNA was fragmented via enzymatic shearing. The resulting fragments were subjected to end-repair, A-tailing, and ligation with indexed adapters. Library fragments were size-selected using AMPure XP beads (Beckman Coulter, Bera, CA, USA). The final library quality was assessed with an Agilent 2100 Bioanalyzer (Agilent Technologies, Santa Clara, CA, USA), and quantification was performed via quantitative PCR (qPCR) [47]. The qualified libraries were sequenced on an Illumina NovaSeq 6000 platform (Illumina, San Diego, CA, USA) to generate 150 bp paired-end reads [48].

Raw sequencing reads were subjected to quality control and adapter trimming using FastQC v0.20.1 with default parameters. This process involved the removal of adapter sequences, low-quality bases (quality score < 20), and reads shorter than 50 bp. The plastome was assembled from approximately 15 million quality-filtered paired-end reads using GetOrganelle v1.7.4.1 [49], a dedicated tool for de novo assembly of organellar genomes. The assembly was performed using a range of k-mer lengths (21, 45, 65, 85, 105) with 15 extension rounds (−R 15) and 100 computational threads (−t 100), employing the embplant_pt (embryophyte plastid) database as a reference. The assembly process began by mapping seed reads to the reference database using Bowtie2 v2.4.1, followed by iterative extension. De novo assembly was subsequently conducted using the integrated SPAdes v3.15.5 algorithm. The final assembly yielded an observed insert size of 237.2 bp (standard deviation: 66.3 bp) and an average plastome coverage of 360.5×.

### 2.3. Plastome Annotation and Visualization

The complete plastome was annotated utilizing a hybrid strategy that integrated both web-based and command-line tools. Initial structural annotation was conducted with GeSeq, leveraging closely related species within the tribe Epidendreae as references to identify protein-coding sequences (CDS), ribosomal RNA (rRNA), and transfer RNA (tRNA) genes [50]. The tRNA predictions were further validated using tRNAscan-SE v2.0 under the organellar genetic code. All gene boundaries and intron-exon structures were manually curated through comparative alignment with homologous genes from reference plastomes and by BLASTp searches (E-value < 1 × 10^−5^) against the NCBI non-redundant protein database. The junctions between the large single-copy (LSC), small single-copy (SSC), and inverted repeat (IR) regions were precisely delineated. A circular graphical map of the genome, illustrating gene arrangement, GC content, and other genomic features, was generated with CPGView [51].

### 2.4. Data Curation and Comparative Dataset Assembly

To establish a robust comparative framework, we retrieved all available plastome sequences for the tribe Epidendreae from the NCBI GenBank database (https://www.ncbi.nlm.nih.gov/genbank/, accessed on 3 February 2025; the species names and accession numbers are listed in Table S1). This initial dataset underwent a stringent quality control pipeline: sequences with less than 95% completeness, poor annotation, or suspected misidentification were excluded. Following this curation process, 39 high-quality plastomes were retained. These were combined with the newly assembled genome *E. tampensis* from this study, resulting in a final dataset of 40 Epidendreae plastomes.

Annotation consistency across all genomes was manually verified using BioEdit v7.2.6 to flag any potential assembly or annotation artifacts [46]. Sequence integrity was further confirmed through BLAST v2.15.0-based verifications. For subsequent evolutionary analyses, protein-coding sequences (CDSs) were extracted from all genomes using custom Perl scripts. These CDS regions were aligned with MAFFT v7.475, and only those sequences with a length that was a multiple of three and without internal stop codons were retained to ensure the reliability of downstream analyses, such as codon usage bias assessment [52].

### 2.5. Analysis of Relative Synonymous Codon Usage

Relative synonymous codon usage (RSCU) was calculated to quantify codon usage bias across the studied genomes. RSCU is defined as the ratio of the observed frequency of a codon to its expected frequency under the assumption of equal usage among synonymous codons. An RSCU value of 1 indicates no bias, values >1 indicate a positive bias, and values <1 indicate a negative bias [53]. For this analysis, we computed RSCU values for all protein-coding sequences in the Epidendreae dataset using custom Python v3.12.2 scripts leveraging the Biopython library. Genes containing internal stop codons or ambiguous nucleotides were excluded. The resulting RSCU matrix was visualized as a heatmap using the pheatmap package in R v4.2.3, with rows representing species and columns representing the 64 codons. Hierarchical clustering was applied to both dimensions to reveal underlying patterns of phylogenetic relatedness and codon usage similarity.

### 2.6. Neutrality Analysis and Evolutionary Forces

To evaluate the influence of mutational pressure versus natural selection on codon usage, we performed a neutrality plot analysis [54]. This approach examines the relationship between the GC content at the first and second codon positions (GC12) and the GC content at the third synonymous position (GC3). Since the third position is often under weaker functional constraint, its nucleotide composition is more reflective of mutational biases, while GC12 is more conserved due to purifying selection on amino acid sequences. For each protein-coding gene, we calculated its average GC12 and GC3 values. The correlation between these variables was assessed using linear regression. The slope of the regression line indicates the relative strength of mutational pressure; a slope close to 1 suggests that mutation dominates the codon usage pattern, whereas a slope near 0 implies a stronger role for natural selection. The coefficient of determination (R^2^) further quantifies the proportion of variance in GC12 explained by GC3, with lower R^2^ values indicating more prominent selective effects. All calculations were implemented in Python, and the results were visualized using ggplot2 in R, with data points colored by corresponding genus to facilitate interpretation.

### 2.7. Analysis of Codon Usage Bias Using ENC–GC3 Plots

The strength of codon usage bias was quantified using the effective number of codons (ENC), as defined by Wright (1990) [55]. ENC values range from 20 (maximum bias, where only one codon is used per amino acid) to 61 (no bias, with all synonymous codons used equally). To evaluate the relative contributions of mutation pressure and natural selection, we compared the observed ENC values against the expected theoretical ENC under a purely GC3-driven mutation model. Genes whose values lie on or near the theoretical curve are considered to be primarily influenced by mutational bias, whereas significant deviations below the curve indicate the action of natural selection.

ENC and GC3 values were computed for all protein-coding genes using Python v3.12.2. The relationship was visualized in scatter plots generated with the ggplot2 package in R, where point size corresponds to ENC value and color represents taxonomic identity at the species level. For clearer phylogenetic interpretation, the data were faceted by genus, allowing direct comparison of codon usage evolutionary patterns across major clades.

### 2.8. Parity Rule 2 (PR2) Plot Analysis

To further dissect the influence of mutational and selection pressures on codon usage, we performed a Parity Rule 2 (PR2) analysis [56]. This method examines the equality in the usage of A versus T (A3/T3) and G versus C (G3/C3) at the third codon position. Under strict mutational equilibrium, points are expected to cluster at the center (0.5, 0.5) of the PR2 plot. Deviations from this point reflect the intensity of directional selection.

We calculated the indices A3/(A3 + T3) and G3/(G3 + C3) for all protein-coding sequences using custom Python scripts. Results were visualized using ggplot2 in R, with a reference grid marking the central unbiased position. Point size was mapped to ENC value, and color was used to distinguish species. A genus-level panel layout was applied to systematically compare bias patterns across taxonomic groups.

### 2.9. Multivariate Analysis of Codon Usage Patterns

To identify major trends in synonymous codon usage, we applied correspondence analysis (COA) to the RSCU matrix using the FactoMineR package in R. This multivariate statistical method reduces the dimensionality of the 59-codon × 40-species dataset into principal axes that capture the dominant variation patterns. The first two principal dimensions, which accounted for the largest proportion of total variance, were retained for visualization and interpretation.

In the resulting COA biplot, each point represents a gene, and its position reflects its specific codon usage profile. Genes with similar codon preferences cluster together, whereas dispersed distributions indicate divergent usage patterns. The visualization was enhanced using the factoextra package, where point size corresponds to ENC value and color denotes species affiliation [57]. To enable taxonomic comparison, the data were faceted by genus, with axis labels explicitly indicating the percentage of variance explained by each component.

### 2.10. Identification and Characterization of Chloroplast Simple Sequence Repeats

Simple sequence repeats (SSRs) in the plastomes of the 40 Epidendreae species were detected using MISA v2.1, with search criteria set as follows: a minimum of 10 repeats for mononucleotides, 6 for dinucleotides, 5 for tri- and tetranucleotides, and 3 for penta- through decanucleotides [58]. Only perfect repeats without interruptions were considered, and homopolymer stretches were excluded to avoid false positives.

Detected SSRs were systematically categorized by: (i) repeat unit length (mono- to decanucleotide), (ii) genomic context (coding sequences, introns, or intergenic spacers), and (iii) copy number. All statistical summaries and visualizations were generated using custom R scripts, providing a comprehensive profile of SSR distribution and composition across the tribe.

### 2.11. Analysis of Evolutionary Selection Pressures (Ka/Ks)

To assess the type and strength of evolutionary selection acting on chloroplast protein-coding genes, we calculated the ratio of non-synonymous (Ka) to synonymous (Ks) substitution rates across all 40 Epidendreae species using a maximum likelihood approach. Protein-coding sequences were extracted and filtered to include only complete, in-frame sequences without internal stop codons. Orthologous gene pairs were identified by gene name correspondence, resulting in 41,790 pairwise comparisons. Sequences were aligned using MAFFT and trimmed with TrimAl to remove poorly aligned regions.

Ka and Ks values were computed using KaKs_Calculator 2.0 with the NG method. Statistical significance of Ka/Ks ratios relative to neutral expectation (Ka/Ks = 1) was assessed using one-sample *t*-tests with Bonferroni correction. Genes were categorized by selection pressure: strong purifying selection (Ka/Ks < 0.5), relaxed purifying selection (0.5 ≤ Ka/Ks ≤ 1), or positive selection (Ka/Ks > 1). Functional categorization followed CPGView classification, with comparisons between categories performed using Wilcoxon rank-sum tests and effect sizes quantified with Cohen’s d. Correlation between Ka and Ks rates within functional categories was assessed using Pearson correlation. All analyses were conducted in R, with outlier pairs (Ka/Ks > 10) excluded to ensure robustness.

### 2.12. Genome Structure and Synteny Analysis

Plastome structure and synteny were analyzed using custom Perl scripts and R packages including genoPlotR, ComplexHeatmap, and circlize [59]. Genome sequences were obtained from GenBank, with gene information including location, strand orientation, and functional category. Genes were color-coded by function: photosynthesis, energy metabolism, transcription/translation, and structural RNAs.

Pairwise synteny was determined using BLASTN (e-value < 1 × 10^−5^), with alignment similarity visualized using a gray gradient [60]. The DNA_seg and comparison functions in R were used to display gene arrangement and collinear connections. Structural analysis included: (1) basic parameters (length, GC content, gene number); (2) regional characteristics (LSC, SSC, IR boundaries); (3) gene composition (protein-coding, tRNA, rRNA); and (4) GC content at different codon positions to assess locus-specific selection effects.

### 2.13. Phylogenetic Reconstruction Using Plastomes

Phylogenetic relationships were reconstructed using 40 Epidendreae complete plastomes. We extracted and aligned protein-coding sequences (nucleotide and amino acid), rRNA, and tRNA genes using MAFFT v7.475, with TrimAl v1.4.rev15 removing poorly aligned regions [52,61].

Three concatenated datasets were analyzed: (i) amino acid sequences for deep relationships, (ii) CDSs for intermediate resolution, and (iii) rRNA sequences for topological validation. Maximum likelihood trees were inferred using FastTree v2.1.11 under JTT + CAT (amino acids) and GTR + CAT (nucleotides) models, with branch support assessed from 1000 bootstrap replicates (support ≥70% considered significant) [62]. Trees were visualized using ggtree in R, integrating genome structural and codon usage data to elucidate Epidendreae evolution within Orchidaceae.

## 3. Results

### 3.1. Plastome Assembly, Structure and Characteristics of Epidendreae

**Figure 1:**
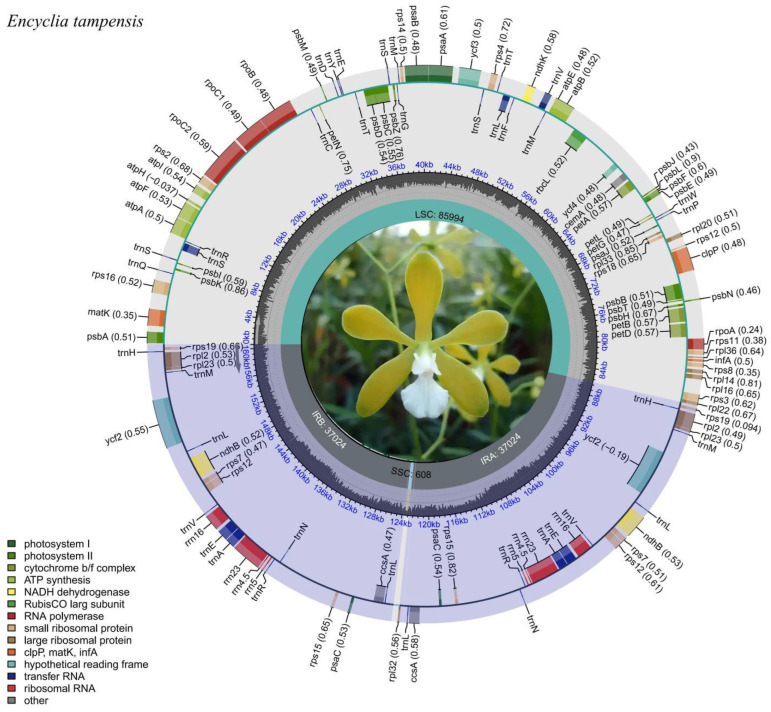
The complete chloroplast genome of *Encyclia tampensis*.

We successfully assembled the complete plastome of *E. tampensis* using a hybrid assembly strategy combining GetOrganelle v1.7.4.1 and NOVOPlasty v4.3.3, yielding a circular genome of 160,650 bp (GenBank Accession = PX501755)with exceptional sequencing quality (mean depth: 482.6×, median: 498×) (Figure 1 and Figure S1A,B). The assembly achieved 100% genome coverage with highly uniform depth distribution across all structural regions (Figure S1C). The interquartile range of the inserted fragment depth was 429.0–554.0×, which approximated a normal distribution (Figure S1B), indicating that technical biases were effectively minimized, thereby ensuring high-quality data, which underpins the reliability of subsequent variant discovery and comparative analysis. The assembled plastome exhibits the canonical quadripartite structure typical of land plant chloroplasts, comprising a large single-copy (LSC) region of 85,994 bp, a small single-copy (SSC) region of 608 bp, and two inverted repeats (IRa and IRb) of 37,024 bp each (Figure 1). Nucleotide composition analysis revealed pronounced AT-rich content, with adenine (A) at 31.15%, thymine (T) at 31.94%, cytosine (C) at 18.69%, and guanine (G) at 18.22% (Table S1). A total of 124 genes were annotated in the *E. tampensis* plastome, including 77 protein-coding genes, 39 tRNAs, and 8 rRNAs, with functional categorization spanning photosystems I and II, ATP synthase, cytochrome b_6_/f complex, and genetic system components (Figure 1 and Table S2). Notably, the plastome demonstrates characteristic IR expansion and pronounced SSC contraction, resulting in the relocation of genes including *trnL* and *ccsA* from the SSC to the IR regions. The overall GC content (36.91%) and positional GC distribution (GC1: 46.96%, GC2: 39.43%, GC3: 30.29%) reflect the AT-biased nucleotide composition prevalent in epidendroid plastomes. Gene structure analysis (Figure S1D) confirmed conserved exon-intron architecture across representative genes, consistent with known Orchidaceae plastome organization and providing a robust foundation for subsequent comparative genomic and phylogenetic investigations.

Comparative analysis of 40 complete plastomes across Epidendreae revealed substantial interspecific variation in genome architecture and nucleotide composition (Table S2). Total genome size varied from 123,455 bp to 160,650 bp, with a mean of 153,112 bp, representing a size range of approximately 37.2 kb. Overall GC content ranged from 36.09% to 37.35%, averaging 36.99%, with positional GC distribution (GC1: 45.30%, GC2: 37.90%, GC3: 29.92%), indicating a moderate AT bias consistent with plastid genomes in epidendroid orchids.

### 3.2. RSCU Analysis

**Figure 2:**
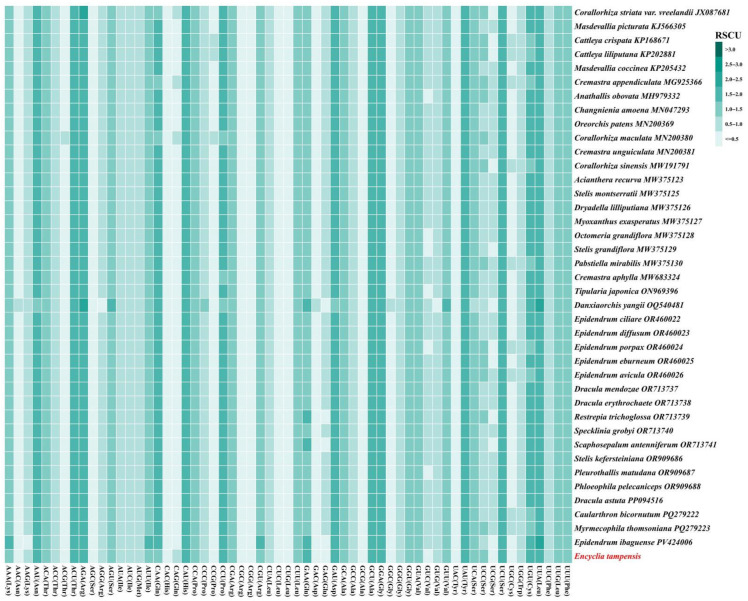
Heatmap visualization of Relative Synonymous Codon Usage (RSCU) patterns across 40 Epidendreae species chloroplast genomes.

We analyzed relative synonymous codon usage across the complete plastomes of 40 Epidendreae species (Figure 2 and Table S2). A consistent pattern of codon usage bias was observed throughout the tribe. Among the 29 codons with RSCU values greater than 1, 16 ended with U, 12 with A, and only 1 with G. This demonstrates that codons terminating in A- or U-terminal nucleotides accounted for 96.55% of all preferred codons (RSCU > 1), significantly outnumbering those ending in adenine (A) or thymine (T) at the third position, while those terminating in guanine (G) or cytosine (C) were underrepresented. This AT-rich bias was observed across all degenerate amino acids, indicating non-random synonymous codon selection in epidendroid plastomes. For leucine, UUA, CUU and UUG were the most frequently used codons, with mean RSCU values of 1.837, 1.258 and 1.219, respectively, whereas CUA, CUC, and CUG showed markedly lower usage. Similarly, for threonine, ACU and ACA were strongly preferred over ACC and ACG, while for valine, GUU and GUA were used significantly more frequently than GUG and GUC. These patterns reflect the overall high AT content of Epidendreae plastomes.

### 3.3. SSR Analysis

**Figure 3:**
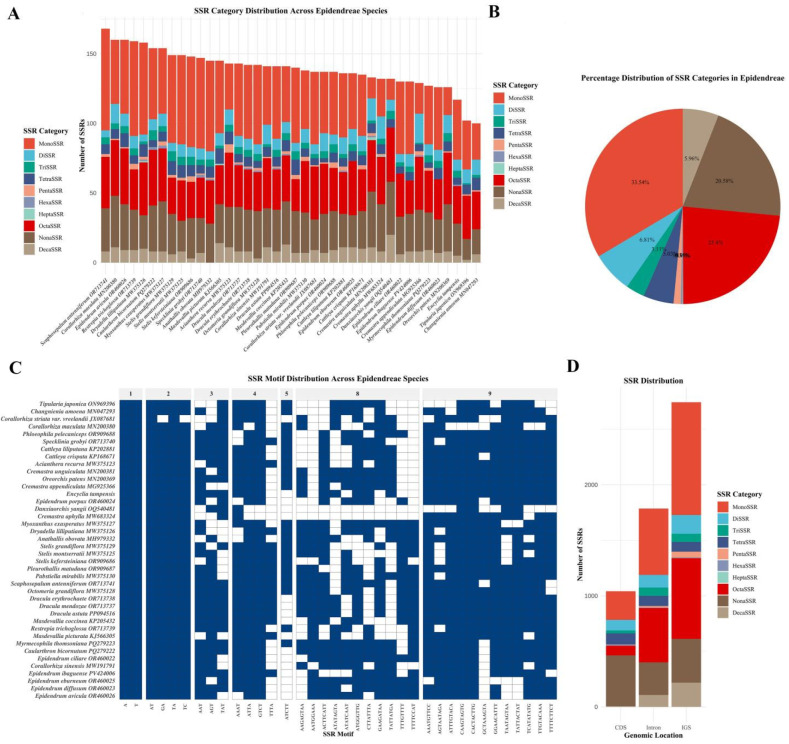
Comprehensive analysis of simple sequence repeats (SSRs) in chloroplast genomes of 40 Epidendreae species. (**A**) Stacked bar chart illustrating the distribution and abundance of SSR categories across the 40 individual Epidendreae species. (**B**) Pie chart showing the percentage distribution of SSR categories identified across the 40 Epidendreae species. (**C**) Heatmap displaying presence/absence patterns of SSR motifs across all 40 analyzed Epidendreae species. (**D**) Distribution of SSR categories across different genomic locations including coding sequences (CDS), intergenic spacers (IGS), and other genomic regions.

Simple sequence repeat (SSR) analysis revealed 5569 perfect microsatellite loci across the 40 Epidendreae plastomes, with individual species harboring 100–168 SSRs per genome (Figure 3A and Table S4A). The distribution of SSR categories revealed an unexpected compositional pattern: mononucleotide repeats (MonoSSR) constituted the most abundant category at 33.54% (1868 loci), followed by octanucleotide repeats (OctaSSR) at 23.4% (1303 loci) and nonanucleotide repeats (NonaSSR) at 20.58% (1146 loci) (Figure 3B and Table S4B). Decanucleotide repeats (DecaSSR) accounted for 5.96% (332 loci), whereas shorter repeat units—dinucleotide (DiSSR, 6.81%), trinucleotide (TriSSR, 3.11%), tetranucleotide (TetraSSR, 5.03%), pentanucleotide (PentaSSR, 1.19%), hexanucleotide (HexaSSR, 0.31%), and heptanucleotide (HeptaSSR, 0.09%)—displayed a decreasing trend. This predominance of longer repeat units contrasts markedly with patterns documented in most angiosperm plastomes, where mononucleotide and dinucleotide repeats typically predominate, suggesting distinctive mutational dynamics operating in Epidendreae plastomes.

Analysis of the genomic compartment distribution revealed pronounced location-specific enrichment patterns (Figure 3D and Table S4C): intergenic spacer (IGS) regions harbored 2742 SSRs (49.24% of the total), with mononucleotide (1014 loci), octanucleotide (727 loci) and nonanucleotide (395 loci) repeats being the most abundant. The intronic regions contained 1786 SSRs (32.07%), exhibiting a similar categorical distribution, whereas the coding sequences (CDSs) harbored only 1041 SSRs (18.69%), with modest variations in the relative frequencies of each category. This pronounced IGS enrichment aligns with the principle that noncoding regions experience relaxed purifying selection relative to protein-coding sequences, permitting greater accumulation of repetitive elements without deleterious functional consequences.

Examination of the SSR motif composition revealed distinct nucleotide bias, reflecting the AT-rich nature of the Epidendreae plastomes (Figure 3C and Table S4D). Among the mononucleotide repeats, adenine (A) and thymine (T) homopolymers overwhelmingly predominated over guanine (G) and cytosine (C) tracts across all 40 species, which is consistent with the tribe’s mean GC content of 36.09% to 37.35%. Highly conserved SSR motifs detected in 100% of the species included simple A and T mononucleotide repeats and AT/TA dinucleotide repeats, along with several nonanucleotide motifs (ATTTGTACA, CAAGTAGTG, CACTACTTG, TATTACTAT, TTGTACAAA) identified in ≥90% of species, suggesting that these represent ancestral repeat elements that have been maintained since subfamily diversification (Figure 3C). Hierarchical clustering analysis based on SSR motif presence/absence patterns revealed strong congruence with established phylogenetic relationships, with congeneric species consistently forming monophyletic clades. For instance, *Dracula astuta*, *D. mendozae*, and *D. erythrochaete* exhibited identical SSR profiles. Notably, exceptions were observed—such as the marked deviation of *Epidendrum porpax* from other *Epidendrum* species—indicating homoplasy in SSR gain/loss events during lineage diversification.

### 3.4. ENC-GC3 Analysis

**Figure 4:**
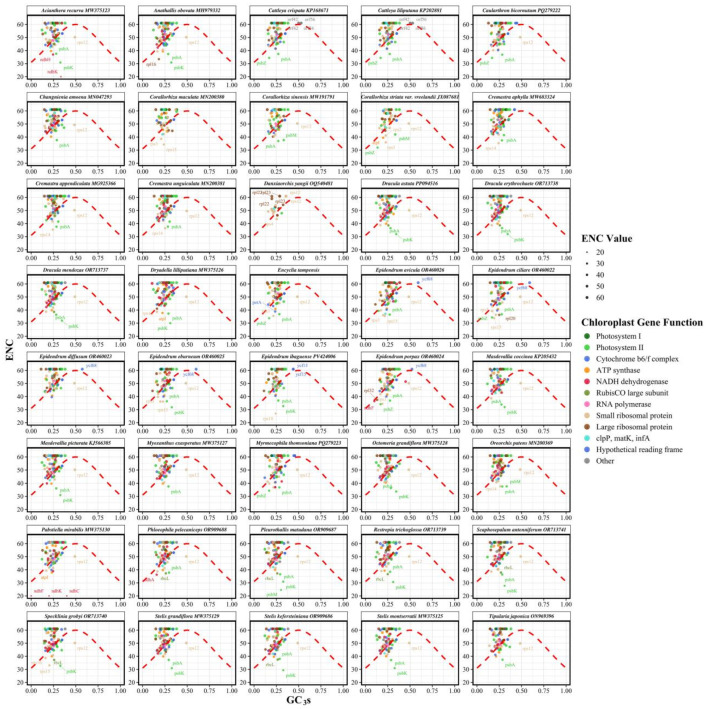
Effective number of codons (ENC) plotted against GC content at synonymous third codon positions (GC3s) for 40 Epidendreae species. Each panel displays species-specific codon usage patterns with individual genes represented as colored points scaled proportionally to ENC values. The theoretical ENC curve (red dashed line) represents the expected relationship under neutral evolution with compositional constraints as the sole evolutionary force.

To evaluate the relative influence of mutational pressure versus natural selection on codon usage patterns, we performed ENC-GC3s analysis. Analysis of 40 Epidendreae species revealed moderate codon usage bias across chloroplast protein-coding genes (Figure 4; Table S5A). The mean ENC values across species ranged from 51.45 in *Pabstiella mirabilis* to 53.34 in *Epidendrum diffusum*, with a tribe average of 52.42 and low interspecific variation. Mean GC3s content also showed limited divergence, ranging from 0.2457 in *Restrepia trichoglossa* to 0.2675 in *Cattleya liliputana*, and averaging 0.2544 across the tribe. The number of analyzed genes per species varied from 25 to 89, reflecting differences in gene complement completeness.

At the gene level, both ENC and GC3s varied substantially, with most genes distributed below Wright’s theoretical curve (Figure 4). This systematic deviation indicates that natural selection, rather than mutational pressure alone, is the principal force shaping synonymous codon usage in Epidendreae plastomes. The pronounced deviation below the curve implies selective pressures related to translational efficiency and functional specificity.

Codon usage bias also varied by functional gene category (Table S5B). Photosystem I genes displayed the weakest bias, indicative of balanced synonymous codon usage. In contrast, the RubisCO large subunit (*rbcL*) exhibited the strongest bias, consistent with intense purifying selection on this key carbon-fixation enzyme. Photosystem II genes showed intermediate but variable bias, while RNA polymerase subunits displayed relatively uniform patterns. GC3 content likewise differed among functional categories (Table S5B). Hypothetical chloroplast reading frames (*ycf* genes) had the highest mean GC3s, whereas cytochrome b6/f complex genes had the lowest. The *rbcL* gene exhibited highly constrained GC3s, aligning with its conserved codon usage. The *ycf* genes showed the broadest GC3s variation, underscoring diverse evolutionary pressures across photosynthetic and genetic components. The consistent distribution of genes below the theoretical ENC-GC3 curve across all functional categories confirms the predominant role of natural selection in maintaining codon usage patterns in Epidendreae plastomes.

### 3.5. Neutrality Analysis

Neutrality analysis across 40 Epidendreae species revealed consistent deviation from neutral evolution in plastome codon usage (Figure S2; Table S6A). The regression slopes between GC12 and GC3 ranged from −0.1865 to 0.1696, with coefficients of determination (R^2^) spanning 0.000–0.0473, demonstrating minimal correlation between nucleotide compositions at different codon positions. Notably, 70% of species exhibited slopes below 0.05, indicating predominant selective constraints overriding mutational pressure. The mean GC3 values clustered within 0.2763–0.2981 (SD: 0.0443–0.0706), while GC12 maintained higher values between 0.4133 and 0.4321 (SD: 0.0496–0.0668), reflecting conserved amino acid composition despite synonymous site variation. Exceptionally, *Cattleya* species (slopes: 0.1660–0.1696) and *Cremastra aphylla* (slope: 0.1473) showed relatively stronger mutational influences, potentially associated with specialized ecological adaptations. The overall compositional conservation and weak GC12-GC3 correlation across phylogenetic diversity support the hypothesis that natural selection—mediated through translational efficiency and functional constraints—acts as the primary evolutionary force shaping synonymous codon usage patterns in Epidendreae plastomes.

Comparative analysis of functional gene categories revealed significant divergence in GC3, GC12, and ENC values across categories, reflecting distinct selective pressures acting on different chloroplast metabolic pathways (Table S6B). The RubisCO large subunit (*rbcL*) exhibited the highest mean GC12 (0.5002) alongside intermediate GC3 (0.2890) and the lowest Effective Number of Codons (ENC = 43.63), indicating strong purifying selection on its amino acid sequence consistent with its critical role in carbon fixation. In contrast, genes encoding the cytochrome b/f complex and ATP synthase showed notably lower GC3 values (0.2589 and 0.2622, respectively), suggesting a greater contribution from compositional mutational pressure. Hypothetical reading frames and other unclassified genes displayed the highest GC3 values (0.3385 and 0.3270, respectively) with greater standard deviations, reflecting relaxed selective constraints and heightened compositional plasticity. Photosystem I and II genes maintained higher mean GC3 (0.3045 and 0.3061) compared to translational machinery components (small ribosomal proteins: 0.2691; large ribosomal proteins: 0.2774), demonstrating greater conservation in third codon-position composition within the photosynthetic apparatus.

### 3.6. PR2 Bias Analysis

Parity rule 2 (PR2) analysis of 3227 protein-coding genes from 40 plastomes across the Epidendreae tribe revealed systematic deviations from neutral equilibrium at synonymous codon positions (Figure S3, Table S7A). The mean G_3_/(G_3_ + C_3_) ratios among species ranged from 0.5311 in *Danxiaorchis yangii* to 0.5527 in *Corallorhiza maculata*, while A_3_/(A_3_ + T_3_) ratios varied between 0.4538 in *Epidendrum avicula* and 0.5001 in *D. yangii*. Members of *Corallorhiza* displayed elevated G_3_/(G_3_ + C_3_) ratios, while most *Epidendrum* species showed reduced A_3_/(A_3_ + T_3_) values. Data points consistently clustered in the lower-right quadrant of the PR2 plot, indicating a universal G-over-C bias and a moderate T-over-A bias across the tribe.

Functional categorization further revealed distinct selective pressures among gene classes (Figure S3, Table S7B). The cytochrome b/f complex and ATP synthase genes exhibited the strongest G-bias (G_3_/(G_3_ + C_3_) = 0.6289 and 0.5800, respectively), whereas Photosystem II genes displayed the most balanced composition (G_3_/(G_3_ + C_3_) = 0.4618). Large and small ribosomal protein genes showed concomitantly elevated G- and A-bias, suggesting a purine-ending codon preference in translation-related genes. Notably, the RubisCO large subunit (*rbcL*) exhibited the lowest ENC value (43.63), consistent with strong purifying selection on this essential carbon-fixing enzyme. Genes with lower ENC values exhibited more pronounced deviations from PR2 parity, reinforcing that natural selection, modulated by gene function and translational, requirements, is the dominant force shaping synonymous codon usage in Epidendreae plastomes, with limited contribution from mutational pressure.

### 3.7. COA

**Figure 5:**
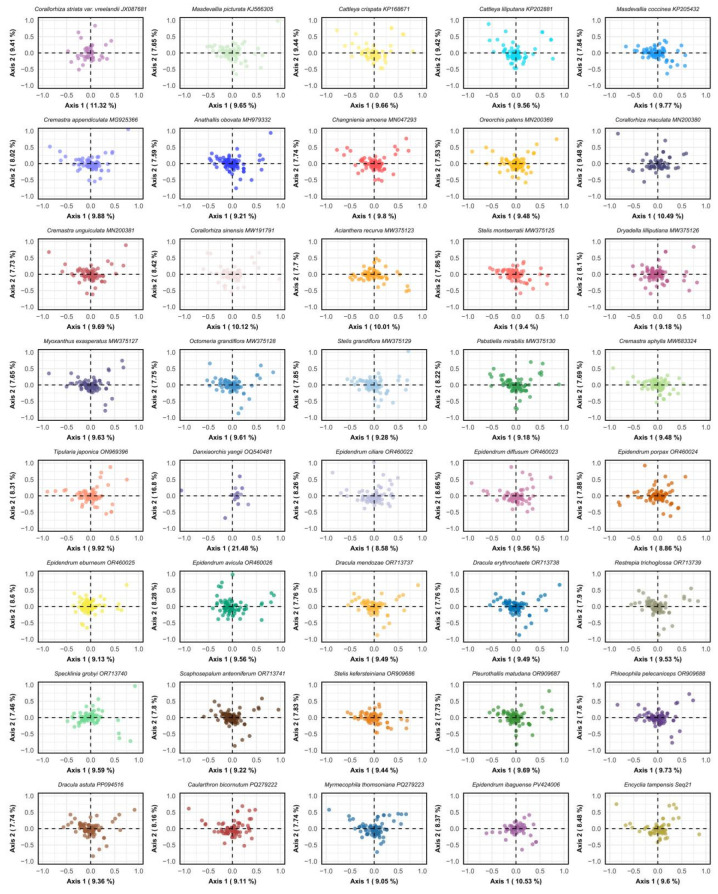
Correspondence Analysis (COA) of synonymous codon usage patterns in 40 Epidendreae chloroplast genomes. Each panel shows the distribution of protein-coding genes in the space defined by the first two COA axes, which capture the primary patterns of codon usage variation. Points represent individual genes, with their positions reflecting similarities and differences in synonymous codon usage patterns.

Correspondence analysis (COA) of 40 Epidendreae plastomes resolved multidimensional patterns in synonymous codon usage variation (Figure 5 and Table S8A). The first two principal axes collectively explained 16.80–30.28% of total variance, with Axis 1 contributing 8.58–21.48% and Axis 2 accounting for 7.46–16.80%. Most species exhibited moderate variance partitioning, though *D. yangii* showed exceptional values (Axis 1: 21.48%, Axis 2: 16.80%), suggesting distinct evolutionary dynamics in this recently diverged lineage. Total inertia ranged from 0.5032 to 0.6257, reflecting substantial heterogeneity in codon usage bias across the tribe. *Corallorhiza* species consistently displayed elevated Axis 1 contributions (10.12–11.32%), while most *Epidendrum* species showed lower values (8.58–10.53%). Species of *Masdevallia* and related pleurothallids exhibited intermediate variance partitioning but lower total inertia, indicating more constrained codon usage evolution in these epiphytic specialists.

Functional gene analysis demonstrated differential representation across categories (Table S8B). Photosystem II and small ribosomal protein genes were universally retained across all 40 species, whereas NADH dehydrogenase genes were absent in three species, confirming sporadic *ndh* gene loss within the tribe. Large ribosomal proteins and core photosynthesis-related genes (e.g., *clpP*, *matK*, *infA*) showed complete conservation, underscoring their essential roles. In the COA ordination space, photosynthetic and translational machinery genes formed tight clusters, consistent with strong functional constraints and purifying selection. In contrast, hypothetical reading frames and “Other” functional categories exhibited broader dispersion, suggesting more relaxed evolutionary constraints.

The moderate cumulative variance explained by the first two COA axes, combined with the wide dispersion of total inertia values, supports a model of multivariate evolution in Epidendreae codon usage. This variation arises from the interplay of mutational biases, selective optimization for translational accuracy and efficiency, gene-specific functional constraints, and phylogenetic history. When integrated with prior ENC-GC3s, neutrality plot, and PR2 analyses, these results confirm that natural selection acts as the dominant force fine-tuning synonymous codon usage, overriding neutral expectations to support adaptive diversification across one of the most species-rich orchid tribes.

### 3.8. Ka/Ks Analysis

**Figure 6:**
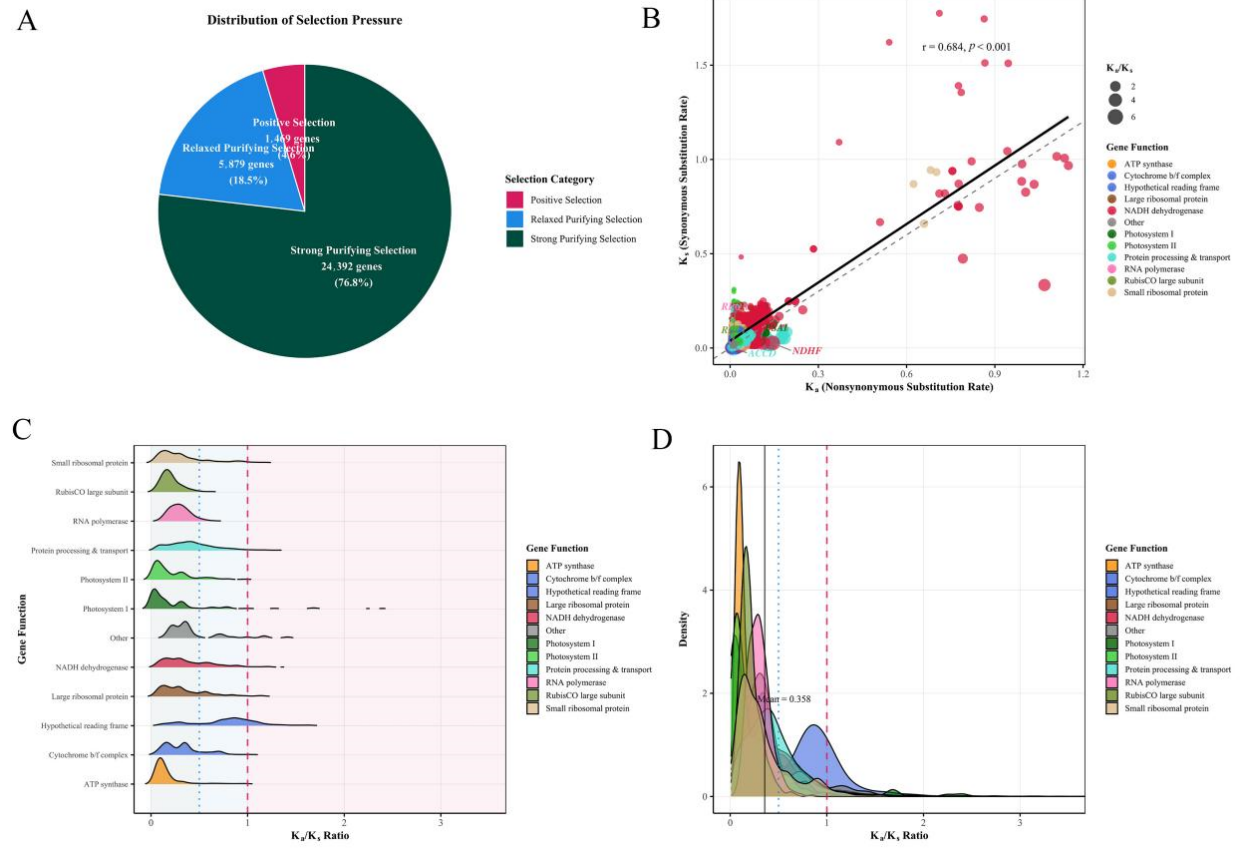
Evolutionary selection pressure analysis of Epidendreae chloroplast genes. (**A**) Pie chart showing the distribution of selection pressure categories across all analyzed gene pairs. (**B**) Scatter plot of Ka versus Ks rates colored by gene functional categories. Point size reflects the Ka/Ks ratio magnitude. (**C**) Ridge density plots showing Ka/Ks ratio distributions across gene functional categories. (**D**) Density curves of Ka/Ks ratios by gene functional categories with the overall mean indicated.

Evolutionary selection analysis of 31,740 gene pairs from Epidendreae plastomes revealed distinct selective constraints across functional gene categories (Figure 6A and Table S9A). Purifying selection predominated across the tribe, with 95.4% of gene pairs exhibiting Ka/Ks < 1.0, of which 76.8% were under strong purifying selection (Ka/Ks < 0.5) and 18.5% under relaxed purifying selection (0.5 ≤ Ka/Ks ≤ 1.0). Only 4.6% of pairs showed Ka/Ks > 1.0, indicating episodic positive selection or relaxed constraints. A Ka versus Ks scatter plot revealed that core photosynthetic genes clustered in the low-Ka, low-Ks region, reflecting strong functional constraint. Regression analysis indicated a positive correlation between Ka and Ks, suggesting that shared mutational processes and generation time effects influence evolutionary rates (Figure 6B).

Selection pressure analysis revealed a hierarchical constraint structure among functional gene classes in the Epidendreae plastomes (Figure 6C,D and Table S9B). The RubisCO large subunit (*rbcL*) exhibited the strongest purifying selection (mean Ka/Ks = 0.205), with no evidence of positive selection found for any gene pairs. Photosystem II (mean Ka/Ks = 0.221) and ATP synthase (mean Ka/Ks = 0.198) genes also exhibited strong functional constraints. In contrast, photosystem I genes showed moderately relaxed selective pressure (mean Ka/Ks = 0.273), with 6.03% of gene pairs under positive selection, suggesting possible adaptive evolution within the light-harvesting complex. RNA polymerase genes maintained moderate purifying selection (mean Ka/Ks = 0.300), while ribosomal proteins exhibited class-specific patterns: Large ribosomal protein (mean Ka/Ks = 0.369) and small ribosomal protein (mean Ka/Ks = 0.351) showed significantly relaxed constraints. Hypothetical reading frames (mean Ka/Ks = 0.813) represented the most permissive selection regime, with 24.46% of gene pairs under positive selection. Bonferroni-corrected Wilcoxon rank-sum tests revealed statistically significant differences in selective constraints between functional gene categories, with particularly striking contrasts between specific gene groups (Table S9C). The most significant evolutionary divergence was detected between hypothetical reading frames and core photosynthetic components, with large effect size differences observed with photosystem II (mean difference = −0.592, Cohen’s d = −1.905), the RubisCO subunit (mean difference = 0.608, Cohen’s d = 1.576), and the ATP synthase (mean difference = −0.615, Cohen’s d = −1.777). RubisCO consistently exhibited the strongest purifying selection in all pairwise comparisons, with statistically significant differences from all functional categories except the “Other” gene category. Ribosomal protein exhibited a hierarchical pattern of constraint, with the large ribosomal protein exhibiting significantly stronger purifying selection than the small ribosomal protein (mean difference = −0.018, Cohen’s d = −0.060). Correlation analysis revealed significant differences in evolutionary dynamics across functional gene classes (Table S9D). NADH dehydrogenase (r = 0.874), RNA polymerase (r = 0.812), and hypothetical reading frame (r = 0.750) showed strong positive correlations, indicating cooperative evolutionary rates, potentially reflecting shared mutational pressure or pleiotropic constraints. In contrast, essential photosynthetic components exhibited weak correlations: Photosystem II (r = 0.177) and ATP synthase (r = 0.150) showed very low Ka-Ks covariation, suggesting decoupled selective pressures between synonymous and nonsynonymous sites. These patterns reflect a fundamental trade-off between functional necessity and evolutionary flexibility—the core photosynthetic apparatus experiences the strongest purifying selection, while genetic system components exhibit greater evolutionary variability. The differential selective pressures across different functional modules of chloroplasts highlight how gene function shapes the molecular evolution of these highly specialized organelles.

### 3.9. Collinearity Analysis

**Figure 7:**
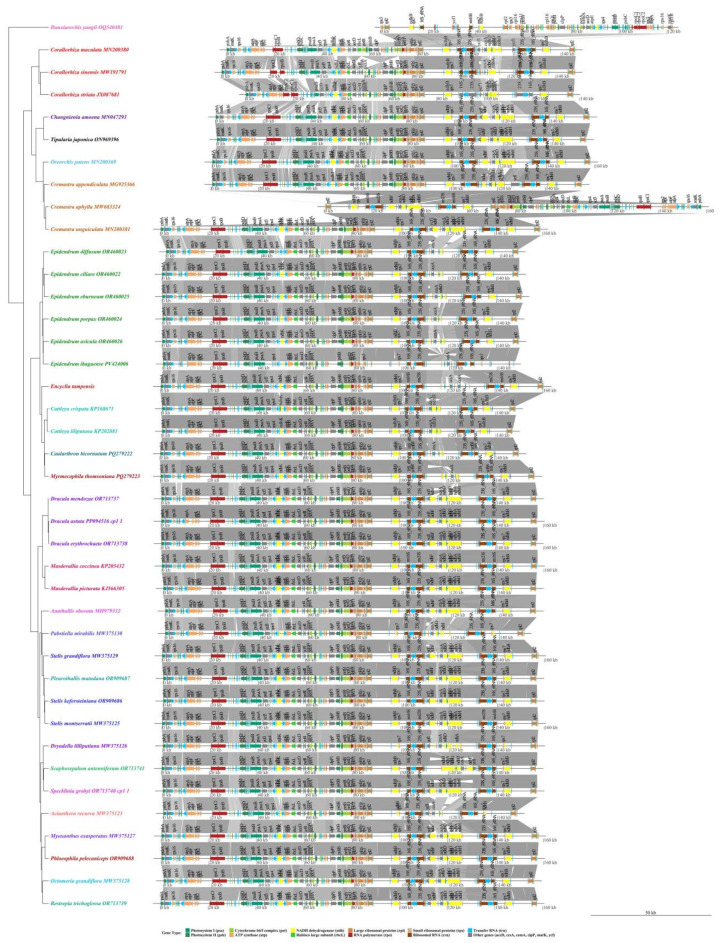
Comprehensive comparative analysis of chloroplast genome organization and gene synteny across 40 Epidendreae species.

Genome-wide synteny analysis of 40 species of Epidendreae revealed a high degree of overall collinearity in their plastid genomes, with species clustering highly consistent with traditional taxonomic boundaries and shared genome structural features (Figure 7). All species examined retained the typical angiosperm quadripartite plastid genome structure, consisting of a large single-copy region, a small single-copy region, and a pair of inverted repeats.

The composition and order of photosynthesis-related genes were highly conserved within the Epidendreae. Core photosynthetic genes (such as *psaA*, *psaB*, and *psbC*) and ATP synthase genes (such as *atpA*, *atpE*, and *atpI*) were remarkably well-positioned and collinear in representative genera such as *Encyclia*, *Epidendrum*, and *Dracula*, suggesting that the arrangement of these core functional genes may have been subject to strong functional constraints during Epidendreae evolution (Figure 7). Furthermore, the RNA polymerase genes *rpoC1* and *rpoC2* maintain a continuous arrangement across all samples, without inversions or deletions. This structural feature is consistent across epiphytic species (e.g., *Encyclia*, *Epidendrum*, and *Dracula*) and saprophytic species (e.g., *Corallorhiza* and *Cremastra*), further demonstrating that the arrangement of genes essential for basic cellular functions is strongly conserved across genera and life forms within the Orchidaceae family. This feature is expected to serve as a potential molecular structural marker for defining taxa within the Orchidaceae family.

With regard to repetitive sequences, the number and distribution of short and long repeats showed significant differences between genera. For example, *Encyclia*, *Epidendrum*, and *Dracula* genera commonly contained core repeat units, and the spacing between these repeats and specific functional genes (e.g., *petB* and *clpP*) was relatively constant (Figure 7). In contrast, *Corallorhiza* lacked long repeat types, and the distribution of these repeats lacked a clear pattern. It is hypothesized that its saprophytic lifestyle may have simplified the repetitive sequences in its genome. Most species exhibited core syntenic blocks of approximately 80 kb in length, while *Caularthron bicornutum* and *Myrmecophila thomsoniana* exhibited distinct 40–60 kb segments containing specific repeat units, significantly different from other groups. This may be due to repeat sequence variation caused by specific contraction events in the genomes of these lineages.

In summary, the syntenic patterns of the plastid genomes of the Epidendreae were closely correlated with their phylogenetic relationships and life forms, reflecting the evolutionary trajectory of the genome under different ecological adaptation contexts.

### 3.10. Phylogenetic Analysis

**Figure 8:**
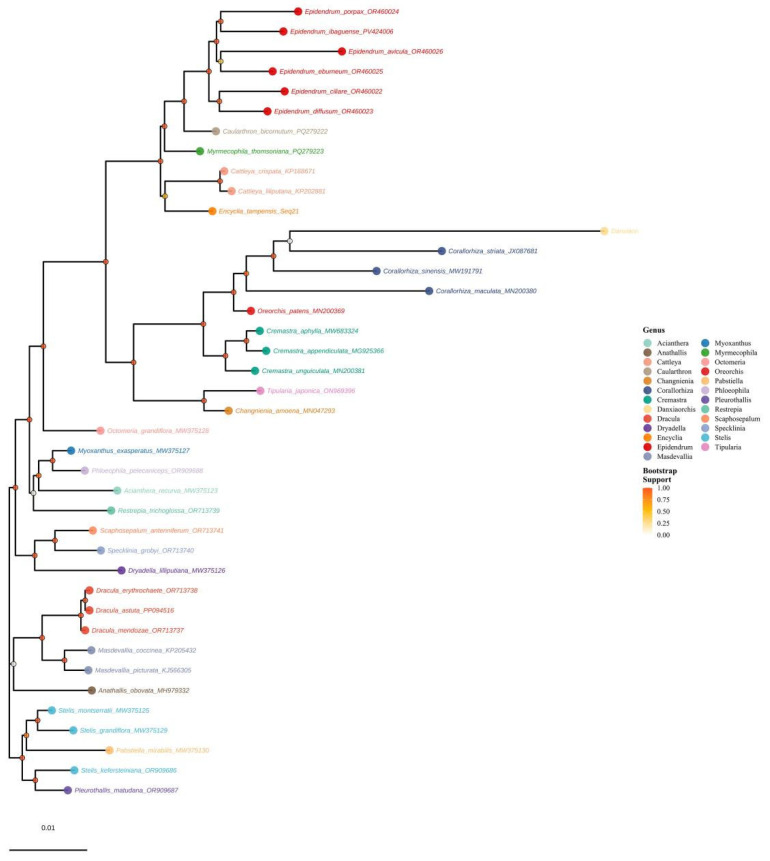
Maximum likelihood phylogeny of Epidendreae based on complete chloroplast proteomes.

Based on a phylogenetic tree constructed from complete plastome protein-coding sequences of 40 species in the Epidendreae, this study robustly resolved the phylogenetic relationships of the major lineages within the tribe. The resulting species clustering was highly consistent with traditional taxonomic boundaries, providing molecular phylogenetic support for the current genus- and subtribe-level divisions based on morphological features (Figure 8). Particularly noteworthy was the high support for the monophyletic grouping of the Pleurothallidinae subtribe (including genera such as *Pleurothallis*, *Stelis*, *Masdevallia*, and *Dracula*) and the Laeliinae subtribe (including genera such as *Epidendrum*, *Cattleya*, and *Encyclia*), confirming the validity of the current classification system.

Branch length analysis revealed significant divergence in evolutionary rates among lineages within the Epidendreae tribe. Among them, the genus *Danxiaorchis* exhibited an extreme evolutionary rate (branch length: 0.04039), suggesting that it may have experienced significant sequence acceleration or possessed a unique life history strategy (Figure 8 and Table S10A). The genus *Corallorhiza* also exhibited a high average branch length (0.01875 ± 0.0052), likely related to its saprophytic lifestyle, which reduces purifying selection pressure on photosynthesis-related genes (Figure 8 and Table S10B). In contrast, genera such as *Epidendrum*, *Encyclia*, *Acianthera*, and *Restrepia* exhibited moderate evolutionary rates (average branch lengths ranging from 0.009 to 0.011). *Dracula* and *Cattleya* exhibited very low branch lengths (averages of 0.00055 and 0.00102, respectively), suggesting strong functional constraints on their chloroplast protein-encoding genes and highly conserved evolutionary processes.

This study systematically revealed the phylogenetic framework of plastomes within the Epidendreae and quantitatively compared the evolutionary dynamics among different lineages. The results suggest that differences in molecular evolutionary rates were closely linked to species’ life forms (such as saprophytic habits) and genomic structural variation, collectively shaping the complex evolutionary paths of the Epidendreae as it adapted to diverse ecological environments.

## 4. Discussion

Through systematic comparative analysis of plastomes from 40 species in the tribe Epidendreae, this study reveals significant structural features, codon usage bias, and phylogenetic relationships within the group’s plastid genomes, providing new insights into the evolutionary mechanisms of this ecologically and taxonomically significant orchid tribe.

### 4.1. Plastid Genome Structural Characteristics and Evolutionary Dynamics

This study systematically documents the structural diversity of plastid genomes in Epidendreae for the first time, particularly identifying multiple cases of extreme structural variation. Plastome sizes in Epidendreae ranged from 123,455 to 160,650 bp. The SSC region of *E. tampensis* was only 608 bp, representing one of the most extreme SSC contractions documented in Orchidaceae, only slightly longer than the previously reported minimum of 524 bp in *Paphiopedilum armeniacum* [63]. This extreme contraction is associated with the transfer of genes typically located in the SSC region, including *trnL* and *ccsA*, to the IR regions. Such IR expansion and SSC contraction patterns have been extensively documented in *Paphiopedilum*, where boundary shifts at the IR/SSC junctions have resulted in highly rearranged SSC regions [63]. The functional implications of extreme SSC contraction remain unclear; however, theoretical considerations suggest that changes in the copy number of genes relocated to the IR region may affect gene expression levels and potentially influence plastome stability during replication [64]. Further experimental studies, including transcriptomic analysis, would be required to assess whether this structural reorganization affects plastome gene expression in *E. tampensis*. This extreme SSC contraction has occurred independently in multiple lineages of Epidendreae, suggesting that it may have important evolutionary significance. Future in-depth studies of these extreme cases will help reveal the biological significance and evolutionary drivers of plastid genome structural variation.

Of particular note, this study demonstrates that the proportion of long repeats, specifically octa- to decanucleotide repeats in the plastid genomes of Epidendreae, is significantly higher than the mononucleotide and dinucleotide repeats traditionally thought to dominate angiosperm plastid genomes. This finding challenges previous paradigms of the distribution of SSRs in plastid genomes [65]. To address potential concerns regarding assembly artifacts, we verified that SSR distributions showed strong congruence with phylogenetic relationships and that read coverage across repeat regions was uniform. The pronounced enrichment of longer repeat units (octa- through decanucleotide repeats) represents an unusual evolutionary pattern warranting investigation into underlying mutational mechanisms, whether arising from replication slippage, recombination-mediated processes, or mobile element activity. Similar to *E. tampensis*, *C. bicornutum* and *M. thomsoniana* also exhibited unique 40–60 kb genomic segments. These structural variations may be associated with their specific epiphytic lifestyles. Further exploration of the distribution patterns of these SSR markers using population genomics approaches has important potential applications in species delineation and conservation genetics [66].

Compared with previous orchid plastome studies, this study demonstrates that genome size variation within Epidendreae is closely associated with life form [67]. Saprophytic lineages such as *Corallorhiza* exhibited significant genome contraction, consistent with the pattern of plastid genome degeneration observed in mycoheterotrophic orchids [68]. In contrast, epiphytic taxa such as *M. thomsoniana* achieved genome expansion through IR region expansion and SSC region contraction. This phenomenon has been systematically documented for the first time within Epidendreae and contrasts interestingly with the patterns observed in the closely related genus *Epidendrum* [43].

### 4.2. Codon Usage Patterns and Evolutionary Drivers

Through multidimensional analyses, this study confirms the dominant role of natural selection in shaping codon usage patterns in Epidendreae plastid genomes, providing new insights into plant plastid genome evolution. Consistent with findings across climatically diverse Theaceae species, Epidendreae plastid genes exhibit moderate codon usage bias, with average ENC of 52.42, and the majority of genes fall below the Wright curve, indicating that selective forces such as translation efficiency optimization outweigh the effects of mutational pressure on codon usage [69]. The RSCU analysis revealed a strong preference for A- and U-ending codons, which is consistent with the AT-rich composition of Epidendreae plastomes. This bias pattern has been observed across diverse orchid lineages and likely reflects both compositional constraints and selection for translational efficiency [63].

Notably, species with extreme SSC contraction, such as *E. tampensis*, exhibited distinct codon usage patterns. Its GC3 content, at 0.254, was below the tribal average, likely related to its highly compacted genome structure. Similar associations have been observed in the genus *Paphiopedilum*, suggesting that major changes in genome structure can have profound impacts on codon usage evolution [70]. Future studies combining transcriptome data will further explore the functional impact of this genomic structural variation on gene expression and adaptive capacity.

Selective pressures differed particularly significantly among functional gene classes. The *rbcL* gene exhibited the strongest purifying selection, with average Ka/Ks = 0.205, and the lowest ENC value of 43.63, consistent with its central function in carbon fixation [71]. This gene encodes the large subunit of RubisCO, the most abundant protein on Earth and the key enzyme responsible for CO_2_ fixation in photosynthesis. The extreme evolutionary constraint observed for rbcL reflects its fundamental importance to plant survival and is consistent with patterns observed across angiosperms [72]. In contrast, hypothetical reading frames exhibited the most relaxed selective pressure, with an average Ka/Ks ratio of 0.813 and 24.46% of gene pairs under positive selection, reflecting the functional diversity of this gene class [73]. These findings, along with previous studies in the genus *Firmiana*, suggest that selective pressure on plastid genes is closely correlated with their functional importance [74].

The protein-coding genes of *E. tampensis* showed patterns of codon usage bias and selective pressure consistent with other epiphytic Epidendreae species. The strong purifying selection observed on photosynthetic genes (*rbcL*, *psbA*, *atpA*) is consistent with the photosynthetic lifestyle of this species, while the more relaxed constraints on *ycf* genes may reflect ongoing pseudogenization or neofunctionalization processes. These molecular evolutionary signatures provide baseline data for understanding how environmental pressures and life history strategies shape plastome evolution in epiphytic orchids.

### 4.3. Phylogenetic Significance and Taxonomic Implications

The phylogenetic tree constructed from complete plastid genome protein-coding sequences strongly supports the major lineage divisions within Epidendreae, providing an important foundation for taxonomic studies of the tribe. The topology was highly consistent with the Orchidaceae classification system established based on multi-gene analyses [75]. Branch length analysis revealed significant heterogeneity in evolutionary rates: *Danxiaorchis* exhibited an extremely high evolutionary rate (branch length 0.04039), possibly related to its unique life history strategy; whereas *Dracula* and *Cattleya* showed strong evolutionary conservatism, reflecting the influence of different life forms on the rate of molecular evolution [68].

Phylogenetic analysis also revealed that species with extreme genomic structural features, such as highly contracted SSC regions, were not monophyletic, suggesting that these features may have evolved independently multiple times within the Epidendreae. For example, *E. tampensis* and *M. thomsoniana* were phylogenetically distant but both exhibited significant SSC contraction. This convergent evolutionary phenomenon warrants further investigation [76]. Expanding the sample base, particularly focusing on rare and endemic taxa, will help to reveal a more comprehensive picture of the evolutionary history of the Epidendreae.

Synteny analysis revealed that the arrangement of core photosynthesis and RNA polymerase genes was highly conserved within Epidendreae, with no rearrangements even between saprophytic and epiphytic groups. This feature can serve as a reliable molecular marker for defining Epidendreae and its internal groups [77]. This finding is consistent with the work on the subtribe Aeridinae, suggesting that the arrangement of certain core genes was fixed early in orchid evolution and has remained highly conserved [78].

### 4.4. Conservation Genetics Implications

The genomic resources generated in this study have direct applications for conservation genetics of Epidendreae species. *E. tampensis*, although one of Florida’s most common epiphytic orchids, faces ongoing threats from habitat loss and commercial exploitation [44]. The microsatellite primers previously developed for this species can be complemented by the plastome-derived markers identified herein for comprehensive population genetic assessments [44]. Specifically, the conserved SSR loci detected across >90% of Epidendreae species offer promising targets for developing universal markers applicable to multiple endangered taxa within the tribe.

The complete plastome sequence of *E. tampensis* enables chloroplast haplotype analysis for assessing population structure and maternal lineage diversity. Such analyses have proven valuable for conservation prioritization in other threatened orchid species [79]. Additionally, the hypervariable intergenic regions identified through our comparative analysis (e.g., those in IGS with high SSR density) provide candidate loci for developing species-specific markers for taxonomic verification and detection of illegal trade in protected orchid species.

The phylogenetic framework established herein facilitates identification of evolutionarily distinct lineages that may warrant special conservation attention. For instance, the genus *Danxiaorchis*, characterized by exceptionally high evolutionary rates, represents a phylogenetically isolated lineage whose conservation may preserve unique evolutionary potential. Integration of our genomic findings with ecological and demographic data will enable more effective conservation strategies for the tribe Epidendreae.

In summary, this study, through multi-faceted comparative genomic analysis, deepens our understanding of the evolution of Epidendreae plastid genomes, particularly with novel findings regarding genomic structural variation, codon usage evolution, and phylogenetic relationships. This study provides solid data support and a robust framework for the systematic classification and evolutionary studies of this important orchid tribe.

## 5. Conclusions

Through systematic comparative analysis of the complete plastomes of 40 species in the Epidendreae, this study makes significant contributions to our understanding of genome structural features, evolutionary mechanisms, and phylogenetic relationships. We systematically reveal for the first time the structural diversity of plastid genomes within this tribe, including significant genome size variation from 123,455 to 160,650 bp, and unique structural features. Most notably, the extreme contraction of the SSC region, which occurred independently in multiple lineages (e.g., an SSC region of only 608 bp in *E. tampensis*), was coupled with a pattern of predominant long repeats that differs from that of typical angiosperms. By integrating multiple methods, including RSCU, ENC-GC3, neutrality plots, PR2, and correspondence analysis, the study confirmed the dominant role of natural selection in shaping codon usage bias and revealed significant differences in selective pressure across functional gene classes, with the *rbcL* gene exhibiting the strongest purifying selection by average Ka/Ks = 0.205 and the *ycf* gene exhibiting the most relaxed selective pressure. The phylogenetic tree constructed from complete plastid genome protein-coding sequences not only strongly supports the traditional classification framework but also reveals significant heterogeneity in evolutionary rates, ranging from rapid evolution in *Danxiaorchis* to extreme conservatism in *Dracula* and *Cattleya*, reflecting the profound influence of different life forms on the rate of molecular evolution. These novel findings provide a new theoretical perspective for understanding the evolution of plastomes in orchids and lay a solid molecular foundation for systematic classification, species identification, and biodiversity conservation in this ecologically and economically important tribe.

## Data Availability

The raw data supporting the conclusions of this article will be made available by the authors on request.

## Supplementary Materials

The following supporting information can be downloaded at: https://www.mdpi.com/article/10.3390/genes16121418/s1, Figure S1: (A) Genome-wide sequencing depth profile; (B) Distribution of sequencing depth; (C) Distribution of coverage categories across the genome; (D) Gene structure diagrams of selected chloroplast genes; Figure S2: Neutrality analysis of codon usage patterns in Epidendreae chloroplast genomes; Figure S3: PR2 plot analysis of codon usage patterns in 40 Epidendreae species chloroplast genomes colored by gene functional categories; Table S1: The species names and accession numbers of the 40 Epidendreae plastomes; Table S2: The statistics of chloroplast genomes from Epidendreae species; Table S3: Relative Synonymous Codon Usage (RSCU) values for all codons across 40 Epidendreae species chloroplast genomes; Table S4A: Species-specific distribution of SSR categories across Epidendreae taxa; Table S4B: Statistical summary of SSR categories in Epidendreae chloroplast genomes; Table S4C: Distribution of simple sequence repeats (SSRs) across genomic locations in Epidendreae chloroplast genomes; Table S4D: Presence/absence matrix of SSR motifs across Epidendreae species; Table S5A: Comprehensive analysis of synonymous codon usage bias in Epidendreae chloroplast genomes: statistical summary of effective number of codons (ENC) and GC content at synonymous third codon positions (GC3s) across 40 species; Table S5B: Functional categorization of chloroplast genes and associated codon usage bias parameters in Epidendreae; Table S6A: Summary statistics of neutrality analysis for Epidendreae chloroplast genomes; Table S6B: Statistical summary by gene functional categories in Epidendreae chloroplast genomes; Table S7A: Statistical summary of codon usage bias analysis for 40 Epidendreae tribe chloroplast genomes; Table S7B: Statistical summary of codon usage bias analysis by gene functional categories in 40 Epidendreae tribe chloroplast genomes; Table S8A: Statistical summary of correspondence analysis for 40 Epidendreae species chloroplast genomes; Table S8B: Statistical summary of correspondence analysis by gene functional categories in 40 Epidendreae species chloroplast genomes; Table S9A: Comprehensive dataset of synonymous and nonsynonymous substitution rates for Epidendreae chloroplast protein-coding genes; Table S9B: Statistical assessment of selective pressures across chloroplast gene functional categories in Epidendreae; Table S9C: Post-hoc pairwise comparisons of dN/dS ratios among functional gene categories using Wilcoxon rank-sum tests with Bonferroni correction; Table S9D: Correlation structure between synonymous (dS) and nonsynonymous (dN) substitution rates within functional gene categories; Table S10A: Species information and phylogenetic characteristics of tribe Epidendreae chloroplast genomes analyzed; Table S10B: Genus-level phylogenetic statistics for tribe Epidendreae chloroplast genome comparative analysis.
