# Supplementary figures and images for: The Complete Chloroplast Genome of Encyclia tampensis (Orchidaceae): Structural Variation and Heterogeneous Evolutionary Dynamics in Epidendreae
Source: Genes (Basel). 2025 Nov 28;16(12):1418. doi: 10.3390/genes16121418 (PMC12733160; doi:10.3390/genes16121418)

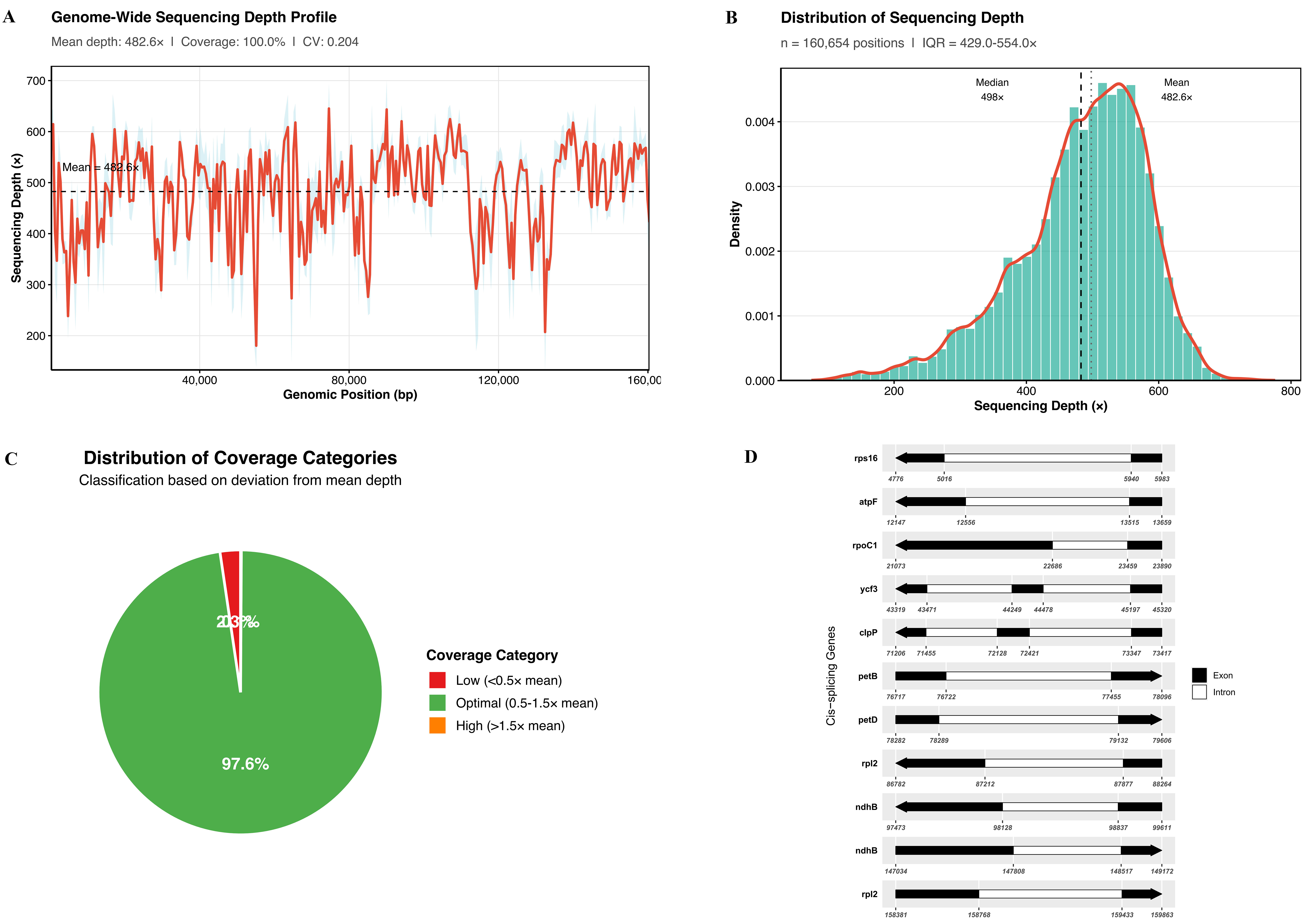

Supplement: Supplementary file 1 [file genes-16-01418-s001.zip › Figure S1 Quality assessment of chloroplast genome sequencing of Encyclia tampensis.jpg]

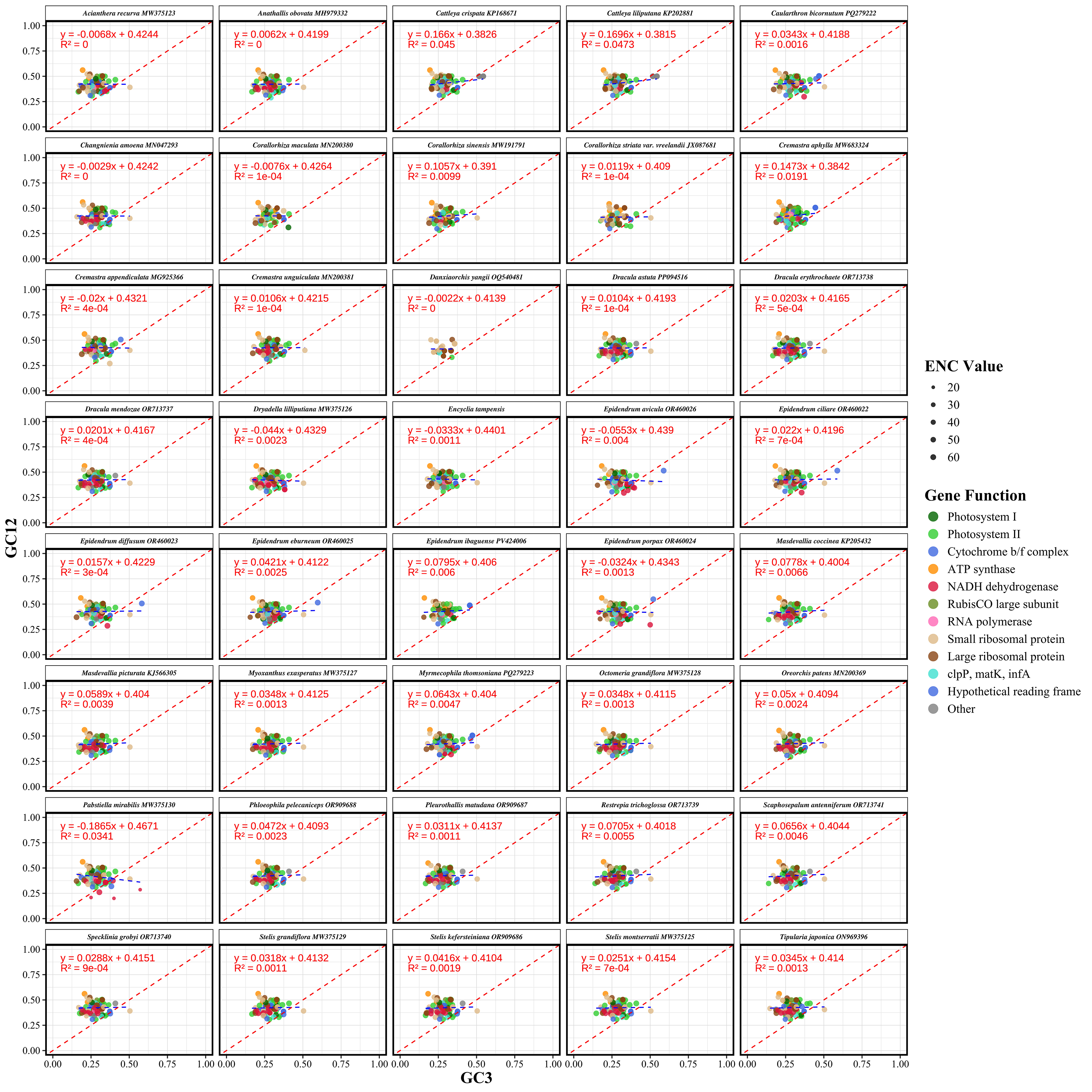

Supplement: Supplementary file 1 [file genes-16-01418-s001.zip › Figure S2 NA of codon usage patterns in Epidendreae cpDNA.jpg]

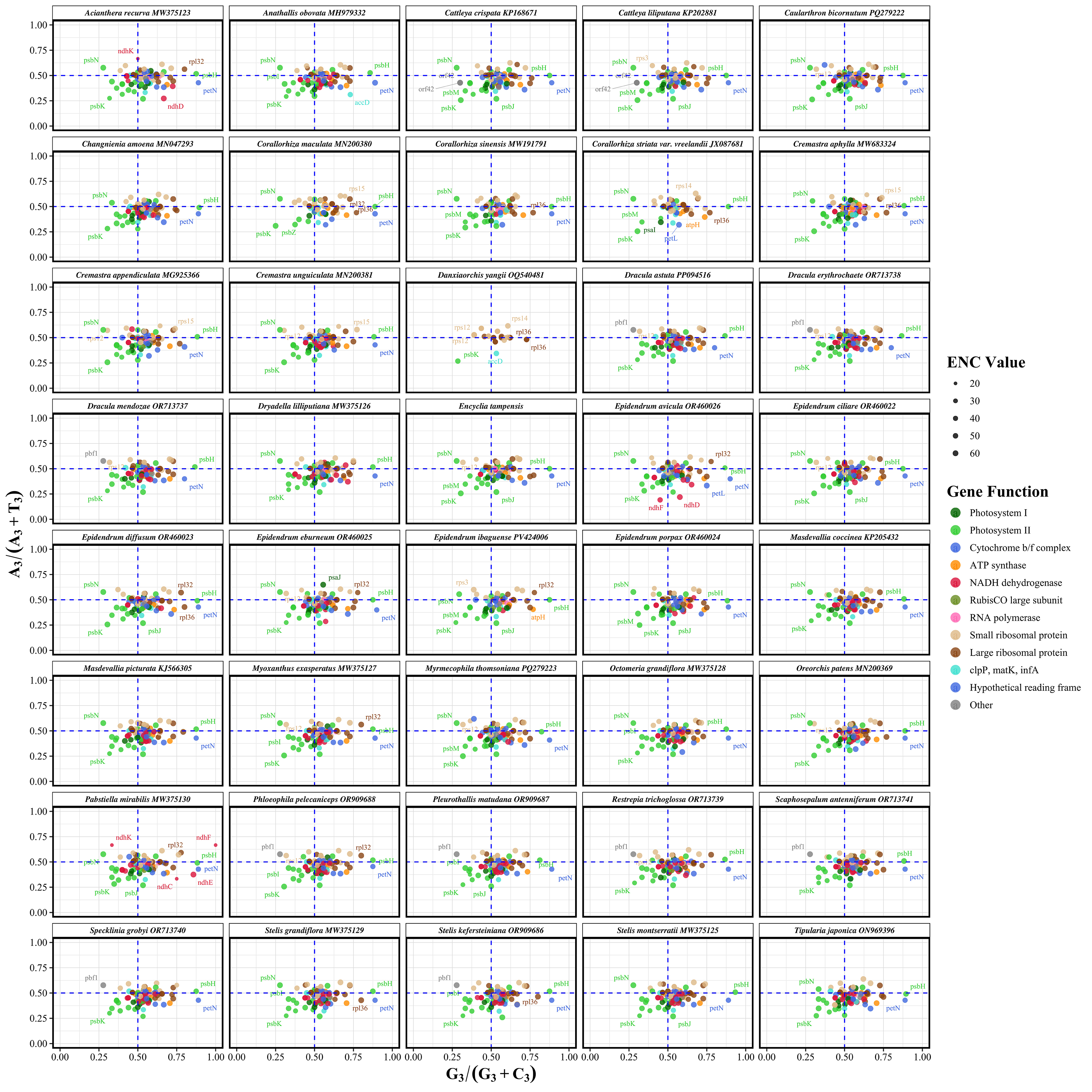

Supplement: Supplementary file 1 [file genes-16-01418-s001.zip › Figure S3 PR2 plot analysis of codon usage patterns in Epidendreae.jpg]
